# Simulating Lagrangian Subgrid‐Scale Dispersion on Neutral Surfaces in the Ocean

**DOI:** 10.1029/2021MS002850

**Published:** 2022-02-05

**Authors:** Daan Reijnders, Eric Deleersnijder, Erik van Sebille

**Affiliations:** ^1^ Institute for Marine and Atmospheric Research Utrecht Utrecht University Utrecht The Netherlands; ^2^ Université Catholique de Louvain Institute of Mechanics Materials and Civil Engineering (IMMC) & Earth and Life Institute (ELI) Louvain‐la‐Neuve Belgium

**Keywords:** Lagrangian, dispersion, diffusion, neutral surfaces, subgrid‐scale, Markov models

## Abstract

To capture the effects of mesoscale turbulent eddies, coarse‐resolution Eulerian ocean models resort to tracer diffusion parameterizations. Likewise, the effect of eddy dispersion needs to be parameterized when computing Lagrangian pathways using coarse flow fields. Dispersion in Lagrangian simulations is traditionally parameterized by random walks, equivalent to diffusion in Eulerian models. Beyond random walks, there is a hierarchy of stochastic parameterizations, where stochastic perturbations are added to Lagrangian particle velocities, accelerations, or hyper‐accelerations. These parameterizations are referred to as the first, second and third order “Markov models” (Markov‐N), respectively. Most previous studies investigate these parameterizations in two‐dimensional setups, often restricted to the ocean surface. On the other hand, the few studies that investigated Lagrangian dispersion parameterizations in three dimensions, where dispersion is largely restricted to neutrally buoyant surfaces, have focused only on random walk (Markov‐0) dispersion. Here, we present a three‐dimensional isoneutral formulation of the Markov‐1 model. We also implement an anisotropic, shear‐dependent formulation of random walk dispersion, originally formulated as a Eulerian diffusion parameterization. Random walk dispersion and Markov‐1 are compared using an idealized setup as well as more realistic coarse and coarsened (50 km) ocean model output. While random walk dispersion and Markov‐1 produce similar particle distributions over time when using our ocean model output, Markov‐1 yields Lagrangian trajectories that better resemble trajectories from eddy‐resolving simulations. Markov‐1 also yields a smaller spurious dianeutral flux.

## Key Points

- We create a 3D isoneutral version of the Markov‐1 Lagrangian dispersion model, similar to Redi's isopycnal rotation of the diffusion tensor
- Dispersion from Markov‐1 includes ballistic and diffusive regimes, making trajectories more realistic than those from random walk models
- Markov‐1 produces a much smaller spurious dianeutral diffusivity than Markov‐0 (random walk)

## 1 Introduction

Turbulent stirring in the ocean disperses tracers and suspended material over time. The eddies, jets, and fronts that characterize this turbulent motion occur at a range of spatial and temporal scales. Since ocean models have a finite resolution, structures with spatial s cales of the order of the grid resolution or smaller are not resolved explicitly. Current state‐of‐the‐art global ocean models use nominal 1/48° grid resolutions (Fox‐Kemper et al., 2019; Su et al., 2018), resolving the mesoscale and part of the submesoscale spectrum. Still, computational constraints limit the simulation length of models at such resolutions to only a few years. Many of the latest generation of Earth system models that are used for CMIP6 use ocean grid resolutions of 1° and 1/4° (Hewitt et al., 2020). The models at 1° do not resolve any mesoscale eddies. While the 1/4° models are eddy‐permitting in parts of the ocean, much higher resolutions are required to resolve the first baroclinic Rossby radius at higher latitudes, such as in the Southern Ocean, where it is O(10 km) (Chelton et al., 1998). Parameterizations of mesoscale eddies therefore remain vital to ocean modeling.

The spreading of tracers due to unresolved eddies is typically parameterized as a diffusive processes, with the evolution of a tracer concentration *C* governed by the advection‐diffusion equation:

(1) $$\frac{\partial C}{\partial t}+\bar{u}\cdot \nabla C=\nabla \cdot \left(K\cdot \nabla C\right),$$

where $\bar{u}$ is the resolved, large‐scale velocity, and **K** is the diffusivity tensor. This practice traces back to Boussinesq's concept of eddy viscosity (Boussinesq, 1877) and G.I. Taylor's work on diffusion (Taylor, 1922), and is still ubiquitous in ocean modeling (Fox‐Kemper et al., 2019). Much research has focused on determining and formulating **K** in order to best represent ocean eddies. This includes aspects like the isopycnal or isoneutral orientation of eddies in the ocean interior (Redi, 1982), their advective effect (Gent & McWilliams, 1990; Griffies, 1998; Haigh et al., 2021), their diffusivity strength (Abernathey et al., 2013; Griesel et al., 2014; Nummelin et al., 2020; Wolfram et al., 2015), and their anisotropy (Bachman et al., 2020; Le Sommer et al., 2011).

Spreading of tracers and suspended material can also be investigated through the Lagrangian framework. Through Lagrangian particle simulations, we can study the pathways of fluid parcels and suspended material forward and backward in time (van Sebille et al., 2018). The Lagrangian framework is an especially useful alternative for the Eulerian framework in studying tracer transport when dealing with point sources (Spivakovskaya et al., 2007; Wagner et al., 2019). Lagrangian simulations use Eulerian ocean model fields to advect virtual particles. This means that Lagrangian simulations also require parameterizations to represent missing dispersion due to the unresolved scales in the Eulerian input data.

The simplest Lagrangian sub‐grid scale dispersion model consists of adding a random walk onto a particle's successive locations. It can be shown that this method is consistent with the advection‐diffusion Equation (1) (Heemink, 1990; Spagnol et al., 2002; Visser, 1997), hence it is often referred to as “diffusion” in Lagrangian literature. It is the simplest member of a hierarchy of stochastic parameterizations that is Markovian in nature, and we will refer to it here as Markov‐0 (Berloff & McWilliams, 2002). “Markovian” relates to the Markov property that each successive displacement in the random walk is independent from the previous.

One shortcoming of Markov‐0 is that, just like the eddy diffusion approximation in Eulerian models, it assumes that eddies have infinitely short time scales. Put differently, it assumes that there is no autocorrelation in the turbulent velocity of the Lagrangian particles. This assumption does not hold true for mesoscale eddies, which transport Lagrangian particles coherently (Berloff & McWilliams, 2002; Haller & Yuan, 2000). Eddy coherence leaves an imprint on the Lagrangian velocity autocorrelation, which can be separated into an exponentially decaying part and an oscillatory part that is the result of phase differences between the eddies and background flow (Klocker, Ferrari, & LaCasce, 2012; Veneziani et al., 2004). Due to this imprint, Markov‐0 is only accurate at time scales when the autocorrelation has decayed away, meaning *t* ≫ *T*_*L*_. Here, *T*_*L*_ is the Lagrangian timescale, equal to the *e*‐folding timescale of the exponential decay of the autocorrelation (LaCasce, 2008). *T*_*L*_ may vary between timescales of a day (Koszalka et al., 2013) to several weeks (see Section 4.2), depending on the characteristics of the ocean domain at hand. If one is concerned with timescales equal to or smaller than *T*_*L*_, Markov‐0 is inadequate for parameterizing subgrid‐scale dispersion. Regardless, this is often the only scheme for parameterizing subgrid‐scale dispersion implemented in community Lagrangian modeling frameworks (van Sebille et al., 2018).

Parameterizations higher in the hierarchy of stochastic models add Markovian noise not on particle locations, but on their velocities (Markov‐1), accelerations (Markov‐2), or even hyper‐accelerations (Markov‐3) (Berloff & McWilliams, 2002; Griffa, 1996; Rodean, 1996; Sawford, 1991). In doing so, these models are capable of better representing dispersion at shorter timescales (for which *t* ≫/ *T*_*L*_), and they can be informed by statistical variances in velocity, acceleration, and hyper‐acceleration, respectively, as well as the timescales over which the autocorrelations of these quantities decay. Further improvements have been formulated that include the looping of particles due to eddy coherence (Reynolds, 2002; Veneziani et al., 2004), as well as the relative dispersion between different particles (Piterbarg, 2002).

Previous ocean applications of this hierarchy of stochastic models in the Lagrangian framework have been restricted to the horizontal plane (e.g. Haza et al. (2007); Koszalka et al. (2013)). However, dispersion through stirring in the interior occurs primarily along sloping surfaces of neutral buoyancy (McDougall, 1987), which are closely related to isopycnals (surfaces of constant potential density). Spivakovskaya et al. (2007) therefore investigated an isopycnal formulation of the random walk dispersion model. Shah et al. (2011) and Shah et al. (2013) further investigated how the spurious diapycnal flux due to numerical integration can best be minimized.

In this study, we discuss, implement, and test an isoneutral formulation of the Markov‐1 subgrid‐scale dispersion model. We compare the Markov‐0 and Markov‐1 models when applied to coarse‐resolution and coarsened model output data. Specifically, we apply these parameterizations to a channel model of the Southern Ocean, with scales and model settings comparable to contemporary global and basin‐scale ocean models. This allows us to also assess the spurious dianeutral flux associated with interpolating discrete ocean model output fields.

Furthermore, we also consider an anisotropic, shear‐dependent formulation of the diffusive/Markov‐0 model, formulated by Le Sommer et al. (2011) (*LS* hereafter), which accounts for anisotropy due to shearing and stretching brought about by mesoscale eddies. Our aim here is to show how one of the many enhancements proposed to the Eulerian diffusion parameterization can be extended to an isoneutral Lagrangian formulation.

This study focuses on how the isoneutral form of the Markov‐1 model, as well as the anisotropic and shear‐dependent form of the Markov‐0 model, can best be implemented, and to which qualitative differences they lead in the dispersion of Lagrangian particles when compared to a dispersionless case and the isotropic Markov‐0 parameterization. We also assess errors of the parameterizations in terms of spurious diffusivities. We aim to use sensible orders of magnitude for the model parameters, but parameter estimation is not our final goal. We are chiefly concerned with formulating an isoneutral form of the Markov‐1 model, laying the groundwork for isoneutral subgrid‐scale Lagrangian models beyond the isotropic diffusive/Markov‐0 parameterization. Higher order stochastic models beyond Markov‐1 and extensions thereof will be left out of the scope of this paper. These should nonetheless benefit from the ideas discussed here. The advective effect of eddies as captured by the Gent‐McWilliams parameterization (Gent & McWilliams, 1990) is also not considered.

In Section 2, we give isoneutral formulations of the Markov‐0 and Markov‐1 parameterizations, as well the anisotropic LS formulation of the Markov‐0 parameterization. Then, in Section 3, we implement and apply these parameterizations to Lagrangian simulations in an idealized situation, and in Section 4 to ocean model data output. We assess the performance qualitatively and quantitatively. Qualitatively, we compare individual particle trajectories and the dispersion of particles in a tracer‐like patch with the dispersion in a fine‐resolution eddy‐resolving model. For the Markov‐1 model we also look at the Lagrangian timescale and associated asymptotic diffusivity, to assess to which extent we can reproduce these profiles in a fine‐resolution setting. Quantitatively, we investigate the spurious dianeutral diffusivity of the different models. These models should keep particles restricted to neutral surfaces, but since we use discrete model output, spurious dianeutral fluxes will occur due to interpolation and other numerical aspects. We wrap up this study with concluding remarks in Section 5.

## 2 Lagrangian Isoneutral Subgrid‐Scale Models

### 2.1 Markov‐0 (Diffusion)

When we interpret the (Eulerian) advection‐diffusion Equation 1 as a Fokker‐Planck equation that gives the probability distribution of particle locations over time (Heemink, 1990), this yields a stochastic differential equation (SDE) describing the evolution of Lagrangian particle positions x as

(2) $$dx=\left[\bar{u}\left(x\right)+\nabla \cdot K\left(x\right)\right]dt+V\left(x\right)\cdot dW\left(t\right).$$

Here, **V** is computed from K as $K=\frac{1}{2}V\cdot V^{T}$, meaning that the random noise on the particle position is proportional to the elements of the diffusivity tensor. This requires **K** to be symmetric and positive‐definite. *d* **W**(*t*) is a vector whose elements correspond to independent Wiener increments in each respective coordinate direction. These Wiener increments are normally distributed random variables N(0,dt) with zero mean and variance *dt* (see also Appendix A from Shah et al. (2011)).

The ∇ · **K**‐term in (Equation (7a), (7b)) ensures the well‐mixed condition (WMC) when the diffusivity tensor is not spatially uniform, and follows the interpretion of Equation 1 as the Fokker‐Planck equation corresponding to the SDE (2) (Heemink, 1990). Simply put, the well‐mixed condition ensures that a particle distribution that is initially mixed, stays mixed. This condition is also essential for the forward‐ and backward‐in‐time formulations of the model to be consistent. The WMC is extensively discussed by Thomson (1987).

The stirring of tracers and dispersion of particles occurs primarily along sloping neutrally buoyant surfaces (McDougall, 1987). Due to uncertainty about its strength, spatial variation, and anisotropy of eddy stirring, the eddy diffusivity is often pragmatically chosen to be a homogeneous and isotropic in the neutral plane, with its strength expressed by the “diffusivity” constant *κ* (with units m^2^  s^−1^). Redi (1982) showed that a diffusivity tensor with these characteristics can be written in geopotential (“*z*‐”) coordinates in terms of the slopes of the locally neutral plane:

(3) $$K_{\text{Redi}}=\frac{\kappa }{1+S_{x}^{2}+S_{y}^{2}}\left[\begin{array}{ccc} 1+\epsilon S_{x}^{2}+S_{y}^{2} & -\left(1-\epsilon \right)S_{x}S_{y} & \left(1-\epsilon \right)S_{x}\\ -\left(1-\epsilon \right)S_{x}S_{y} & 1+S_{x}^{2}+\epsilon S_{y}^{2} & \left(1-\epsilon \right)S_{y}\\ \left(1-\epsilon \right)S_{x} & \left(1-\epsilon \right)S_{y} & \epsilon +S_{x}^{2}+S_{y}^{2} \end{array}\right],$$

where *ϵ* ≡ *κ*_dia_/*κ* denotes the ratio of dianeutral (diabatic) to isoneutral diffusivity, and *S*_*x*_ and *S*_*y*_ are the slopes of the neutral surfaces. When the neutral surfaces are aligned with the isopycnals, which is the case for an equation of state that is linear in salinity and potential temperature, these slopes are found as

(4) $$S_{x}=-\frac{\partial \rho }{\partial x}/\frac{\partial \rho }{\partial z},\;S_{y}=-\frac{\partial \rho }{\partial y}/\frac{\partial \rho }{\partial z}.$$

Cox (1987) showed that the diffusivity tensor (3) can be simplified when these slopes are small (say $|S|=\sqrt{S_{x}^{2}+S_{y}^{2}}<10^{-2}$, which is generally the case in the ocean), and when *ϵ* is small compared to unity, so that it reduces to

(5) $$K_{\text{Redi},\text{approx}}=\kappa \left[\begin{array}{ccc} 1 & 0 & S_{x}\\ 0 & 1 & S_{y}\\ S_{x} & S_{y} & \epsilon +|S{|}^{2} \end{array}\right].$$

Particle trajectories can then be computed by integrating Equation 2. A *κ* that is constant in space and time corresponds to the idealized case of homogeneous and stationary turbulence. The model has the Markovian property that successive spatial perturbations **V** · *d* **W**(*t*) are uncorrelated. This in turn causes successive particle velocities $v=\frac{\partial x}{\partial t}$ to be uncorrelated as well, which is unrealistic at short timescales (i.e., *t* ≫/ *T*_*L*_; LaCasce, 2008).

### 2.2 Anisotropic Shear‐Dependent Markov‐0

While the tensors (3) and (5) assume that the diffusivity is isotropic and uniform in the isoneutral plane and time, the transport and stirring by eddies leads to effective diffusivities that are highly inhomogeneous and anisotropic (McWilliams et al., 1994; Nummelin et al., 2020; Sallée et al., 2008). In ocean modeling, the effects of eddies on momentum transfer are represented by an eddy viscosity. To account for the inhomogeneous effect of eddies on the momentum transfer, the eddy viscosity is often parameterized using the Smagorinsky parameterization (Smagorinsky, 1963), which relates the strength of the viscosity to the local shear of the flow based on closure of the momentum equations. This parameterization can also be used for tracer diffusion (Le Sommer et al., 2011), and has been applied for spatially‐dependent (horizontal) random walk dispersion to parameterize eddies in Lagrangian studies (Nooteboom et al., 2020).

Le Sommer et al. (2011) derived an anisotropic and shear‐dependent diffusion parameterization, related to the Smagorinsky parameterization, that also accounts for the anisotropy in effective diffusivity due to the shearing and stretching effect from the resolved scales on the unresolved scales. This parameterization, here abbreviated as *LS*, was originally proposed for parameterizing the submesoscale using resolved mesoscale motions, but Nummelin et al. (2020) suggest that the LS parameterization can be applied to coarser models in which the mesoscale is not resolved.

The isoneutral diffusivity tensor from the LS parameterization is given by

(6) $$K_{\text{LS}}=\frac{h^{2}}{2}\left(1+\delta^{2}\right)\left[\begin{array}{ccc} p & r & pS_{x}+rS_{y}\\ r & q & rS_{x}+qS_{y}\\ pS_{x}+rS_{y} & rS_{x}+qS_{y} & pS_{x}^{2}+qS_{y}^{2}+2rS_{x}S_{y} \end{array}\right],$$

with $p=\sqrt{r^{2}+a^{2}}+a$ and $q=\sqrt{r^{2}+a^{2}}-a$. Here, $r=\frac{\partial v}{\partial x}+\frac{\partial u}{\partial y}$ is the rate of shear strain and $a=\frac{\partial u}{\partial x}-\frac{\partial v}{\partial y}$ the rate of normal strain, both in the horizontal plane. The underlying assumption is that the largest contribution to the isoneutral dispersion falls within the horizontal plane. The *h*‐term is the horizontal filter size over which the parameterization acts, and $\delta =\left[\frac{\partial u}{\partial x}+\frac{\partial v}{\partial y}\right]/\sqrt{r^{2}+a^{2}}$ is a non‐dimensional divergence parameter. The filter size *h* is related to the size of the grid and it should be tuned through an *O* (1), model‐dependent constant *C* that depends on the underlying advection scheme, so *h* ^2^ = *C*dx ⋅ *dy*. A fixed dianeutral diffusivity *ϵκ* can be set if we approximate it as a vertical diffusivity and add it to **K**_LS,33_.

This parameterization can readily be used in Lagrangian simulations by using **K**_LS_ (6) for the Markov‐0 model (2). The parameterization is inherently local, with each of the parameters computed on the location a Lagrangian particle (or grid cell, in the Eulerian case).

### 2.3 Markov‐1

Next in the hierarchy of stochastic subgrid‐scale dispersion models is the Markov‐1 model, also known as the random acceleration or Langevin model (Berloff & McWilliams, 2002). The Markov‐1 model adds a random forcing on particle velocities, which should be proportional to the velocity variance associated to the unresolved eddies. The model's governing equations are.

(7a) $$dx=\left[\bar{u}\left(x\right)+u^{\prime }\right]dt,$$

(7b) $$du^{\prime }=\left[-\left[\theta^{-1}\left(x\right)\right]\cdot u^{\prime }+\tilde{a}\left(x,u^{\prime }\right)\right]dt+b\cdot dW\left(t\right).$$

The particle location **x** evolves through integration of the resolved mean flow $\bar{u}\left(x\right)$ and a turbulent fluctuation **u′**. This fluctuation evolves through the stochastic differential Equation (7b). The deterministic part of this equation consists of two terms: a fading‐memory term, which ensures an exponential decay in the autocorrelation of the particle's velocity, regulated through the fading‐memory time tensor ***θ*** (with time as its dimension), and a drift correction term $\tilde{a}$, which ensures the well‐mixed condition. The stochastic forcing term consists of the Wiener increment *d* **W** and the random forcing is related as ***b** **b*** ^*T*^ = 2 ***σθ*** ^−1^. Here, ***σ*** is the velocity variance tensor, which relates to the strength of the velocity fluctuations u**′** that are to be simulated:

(8) $$\sigma_{ij}=\langle u_{i}^{\prime }u_{j}^{\prime }\rangle ,$$

where the angled brackets denote ensemble averages over Lagrangian trajectories.

The drift correction term is given by

(9) $${\tilde{a}}_{i}=\frac{1}{2}\frac{\partial \sigma_{ik}}{\partial x_{k}}-\frac{\sigma_{im}}{2}\left({\bar{u}}_{k}+u_{k}^{\prime }\right)\frac{\partial \left[\sigma^{-1}\right]}{\partial x_{k}}u_{j}^{\prime }.$$

See Berloff and McWilliams (2002) for further details and derivations.

The nonsingular velocity variance tensor ***σ*** and the fading‐memory time tensor ***θ*** are the free parameters in the Markov‐1 model. They can be estimated from velocity fields in which the turbulent velocity is resolved. For the velocity variance, this is clear from Equation 8. The velocity variance tensor may be anisotropic, inhomogeneous in space, and evolving over time. An obvious and useful simplification is to use a single, average velocity variance parameter *ν* ^2^ that characterizes the entire system (Koszalka et al., 2013). In this case *σ* is diagonal with its values equal to *ν* ^2^. Alternatively, the velocity variance may be a probability distribution rather than an average value in order to account for the variance in *ν* ^2^ found within different regions of a fluid domain (Berloff & McWilliams, 2003).

The fading‐memory time tensor ***θ*** determines the strength of the exponential decay of the turbulent velocity **u′**. The elements of ***θ*** are found by integrating the Lagrangian autocorrelation *R*_*ij*_(*τ*) over all time lags *τ*:

(10) $$\theta_{ij}=\int_{0}^{\infty }R_{ij}\left(\tau \right)d\tau ,$$

where

(11) $$R_{ij}\left(\tau \right)=\langle u_{i}^{\prime }\left(t\right)u_{j}^{\prime }\left(t+\tau \right)\rangle /{\left(\langle u_{i}^{\prime 2}\rangle \;\langle u_{j}^{\prime 2}\rangle \right)}^{1/2}.$$

Like the turbulent velocity, the Lagrangian autocorrelation exhibits spatial variation in the ocean, and its anisotropy can be strongly affected by the presence of jets (Griesel et al., 2010). Still, it is also useful to characterize the fading‐memory time of the entire system by an average value. In a homogeneous, stationary situation without boundary effects, the fading memory tensor is diagonal with its values equal to the Lagrangian integral time *T*_*L*_.

We characterize the dispersion of particles by the single‐particle (sometimes called “absolute”) dispersion tensor:

(12) $$D_{ij}(t,x(0))=\langle (x_{i}(t)-x_{i}(0))(x_{j}(t)-x_{j}(0))\rangle .$$

Berloff et al. (2002) note that while the dispersion tensor in the ocean may evolve in a nonlinear manner, it can be described by different power laws at intermediate timescales:

(13) $$D_{ii}\left(t\right)∼t^{\alpha_{ii}}.$$

Single‐particle dispersion in the ocean is initially ballistic, meaning *D*(*t*) ∼ *t* ^2^ for *t* ≪ *T*_*L*_. At longer time‐scales, it becomes approximately linear in time, that is, *D*(*t*) ∼ *t*. Since such behavior is equivalent to that of a diffusive process, this is also referred to as the diffusive limit. Unsurprisingly, dispersion simulated by the Markov‐0 model is purely diffusive. The Markov‐1 model, however, is able to also simulate the initially ballistic behavior of particles dispersion. For time scales longer than those characterized by the elements of ***θ***, the Markov‐1 model essentially behaves diffusively (Rodean, 1996). In this limit, assuming homogeneity, stationarity, and absence of boundary effects, we can relate the absolute diffusivity, velocity variance and Lagrangian integral time as

(14) $$\nu^{2}T_{L}=\kappa .$$

At intermediate time‐scales, *α*_*ii*_ can take on other values than 1 and 2, which is referred to as anomalous dispersion (LaCasce, 2008). While the dispersion regimes other than the ballistic and diffusive cannot be simulated by Markov‐1, the higher order Markov‐2 and Markov‐3 models, or modifications of Markov‐1 are able to account for such behavior, such as the oscillatory component of the Lagrangian autocorrelation (Berloff & McWilliams, 2002; Reynolds, 2002; Veneziani et al., 2005). However, we limit ourselves here to Markov‐1 for its simplicity, as each modification or higher model in the hierarchy includes more free parameters.

We now formulate an ad‐hoc three‐dimensional, isoneutral version of the Markov‐1 model in the case of homogeneous and stationary turbulence without boundary effects. First, we assume that the turbulent velocity perturbations should remain primarily restricted to the local neutral plane, in which it is isotropic. In isoneutral coordinates this yields

(15) $$\sigma_{\text{iso}}=\left[\begin{array}{ccc} \nu^{2} & 0 & 0\\ 0 & \nu^{2} & 0\\ 0 & 0 & \eta \nu^{2} \end{array}\right],\;and\;\theta_{\text{iso}}=\left[\begin{array}{ccc} T_{L} & 0 & 0\\ 0 & T_{L} & 0\\ 0 & & \varepsilon T_{L} \end{array}\right].$$

Assuming there is some dianeutral velocity perturbation $\nu_{\text{dia}}^{2}$ (≪*ν* ^2^), we define $\eta \equiv \nu_{\text{dia}}^{2}/\nu^{2}$. Similarly, assuming a separate dianeutral Lagrangian integral time *T*_*L*,dia_, we define *ɛ* ≡ *T*_*L*,dia_/*T*_*L*_.

Then, we simply transform ***σ*** and ***θ*** from isoneutral coordinates to geopotential coordinates in analogy to Redi's formulation of the isoneutral diffusivity tensor (Redi, 1982). This yields:

(16) $$\sigma_{\text{geo}}=\frac{\nu^{2}}{1+S_{x}^{2}+S_{y}^{2}}\left[\begin{array}{ccc} 1+\eta S_{x}^{2}+S_{y}^{2} & -\left(1-\eta \right)S_{x}S_{y} & \left(1-\eta \right)S_{x}\\ -\left(1-\eta \right)S_{x}S_{y} & 1+S_{x}^{2}+\eta S_{y}^{2} & \left(1-\eta \right)S_{y}\\ \left(1-\eta \right)S_{x} & \left(1-\eta \right)S_{y} & \eta +S_{x}^{2}+S_{y}^{2} \end{array}\right],$$

and

(17) $$\theta_{\text{geo}}=\frac{T_{L}}{1+S_{x}^{2}+S_{y}^{2}}\left[\begin{array}{ccc} 1+\varepsilon S_{x}^{2}+S_{y}^{2} & -\left(1-\varepsilon \right)S_{x}S_{y} & \left(1-\varepsilon \right)S_{x}\\ -\left(1-\varepsilon \right)S_{x}S_{y} & 1+S_{x}^{2}+\varepsilon S_{y}^{2} & \left(1-\varepsilon \right)S_{y}\\ \left(1-\varepsilon \right)S_{x} & \left(1-\varepsilon \right)S_{y} & \varepsilon +S_{x}^{2}+S_{y}^{2} \end{array}\right].$$

Note that in order for these tensors to be nonsingular, *η* and *ɛ* should be nonzero, meaning that ***σ***_geo_ and ***θ***_geo_ have nonzero diapycnal contributions. We thus have to specify *η* and *ɛ* in a way such that they are small enough to prevent large dianeutral excursions.

While the diffusivity tensor (Equation (7a), (7b)) can be simplified (Equation (7a), (7b)) by the assumption that slopes are small, this assumption cannot be applied to the tensors ***σ***_geo_ (Equation (7a), (7b)) and ***θ***_geo_ (Equation 17), since the terms that are scaled out in the small‐slope assumption become dominant in the inverses of ***σ***_geo_ and ***θ***_geo_, which are used in Equation (7a), (7b) and Equation (7a), (7b) and when computing ***b***.

A key assumption of Redi's diffusivity tensor **K**_redi_ is that the neutral surfaces are stationary and locally flat. “Locally” here is related to the length scale associated to the displacement of a particle over one timestep. The assumption is that when a particle is advected, the neutral slope at the particle's original location **x**_0_ at time *t*_0_ is approximately equal to the neutral slope at the particle's new location **x**_1_ after a timestep *dt*. Any difference in the orientation of the neutral surface over successive timesteps will lead to some dianeutral movement, but as long as neutral surfaces are locally flat, this dianeutral movement is limited and the new local slopes are used for computing the next neutral displacement.

For Markov‐1, the situation is more complicated. In this case, the stochastic velocity perturbations of a particle at time *t*_0_ and location **x**_0_ are oriented parallel to the local neutral plane. However, since particle velocities (Equation (7a), (7b)) are autocorrelated, the curvature of the neutral surface at a particle's initial location **x**_0_ can influence a particle's velocity over several timesteps, as the particle is displaced away from **x**_0_. This influence decays exponentially with the *e*‐folding timescale *ɛT*_*L*_. Thus if a neutral surface curves at spatial scales that are similar to or smaller than the length scale *L* over which a particle travels within the timescale *ɛT*_*L*_, the signal of the turbulent velocity perturbation at *t*_0_ influences the particle's net turbulent velocity, causing a dianeutral velocity contribution, and therefore a dianeutral displacement. To combat this dianeutral movement, the Lagrangian autocorrelation in the dianeutral direction should rapidly decay away at each timestep. Put differently, *ɛT*_*L*_ should be so small that a neutral surface can be approximated as flat over the length scale *L*. While *ɛT*_*L*_ should be larger than zero to avoid singularity of ***θ***, one ad‐hoc workaround to rapidly extinguish the signal of velocity perturbations at previous timesteps is to set

(18) $$\varepsilon T_{L}=dt.$$

This workaround comes at a price: if the neutral surface curves, the Lagrangian decorrelation of an initially isoneutral signal may occur more quickly than is prescribed by ***θ***, since the initially isoneutral perturbation becomes dianeutral over time, which causes it to decay rapidly due to (18). This effect increases when more curvature is covered by a Lagrangian particle as it moves in space and time. Properly retaining autocorrelations on curved surfaces is a complicated matter (Gaspari & Cohn, 1999), so here we take a pragmatic approach by assuming that the change in isoneutral curvature is small enough for practical use to warrant our ad‐hoc formulation of a three‐dimensional Markov‐1 model.

Finally, when *ɛ* is fixed by Equation (7a), (7b), *η* can be chosen in such a way that the effective dianeutral diffusivity in the limit *t* ≫ *T*_*L*_ is controlled as:

(19) $$\epsilon \kappa =\eta \;\nu^{2}\;\varepsilon \;T_{L}.$$

This means that if we indeed assume homogeneity, stationarity, and a lack of boundary effects, the parameters necessary for Markov‐1 model may be determined by specifying the Lagrangian integral time *T*_*L*_ and an effective diffusivity *κ*, which fix *ν* ^2^ through Equation 14, and by specifying the dianeutral diffusivity ratio *ϵ*, fixing *ɛ* and *η* (through Equation 18 and Equation 19).

## 3 Numerical Implementation

### 3.1 Discretization

To use the Markov‐0 and Markov‐1 models numerically, we need to discretize SDEs Equation (7a), (7b) and Equation (7a), (7b). The simplest SDE discretization is Euler‐Maruyama scheme, which can be seen as a stochastic version of the Euler‐forward scheme. Given a general stochastic differential equation

(20) dX=α(X,t)dt+β(X,t)dW(t),

with *α*(**X**, *t*) signifying the deterministic forcing strength and *β*(**X**, *t*) the stochastic forcing strength, the Euler‐Maruyama scheme approximates the true solution for ***X*** by the Markov chain ***Y*** as

(21) $$Y_{n+1}^{k}=Y_{n}^{k}+\alpha^{k}\Delta t+\sum_{j=1}^{m}\beta^{k,j}\Delta W^{j},$$

where superscripts denote the *k*th component of the *m*‐dimensional vectors **X** and **Y** and subscripts denote discrete time indices. Δ**W** is an *m*‐dimensional vector of discretized Wiener increments, which are normally distributed, N(0,Δt), with zero mean and variance Δ*t*. See Kloeden and Platen (1999) or Iacus (2008) for more details on numerical SDE schemes. The expressions for *α* and *β* can be readily identified in Equation (7a), (7b) and Equation (7a), (7b). In the case of Markov‐1, an additional numerical integration is necessary for Equation (7a), (7b). For consistency with the Euler‐Maruyama scheme, this can simply be the Euler‐Forward discretization.

We implemented the Markov‐0 and Markov‐1 schemes in the Parcels Lagrangian framework (Delandmeter & van Sebille, 2019). All Lagrangian simulations in this paper are carried out with Parcels (van Sebille et al., 2020).

### 3.2 Idealized Test Case

We assess the validity of the isoneutral subgrid‐scale models using an idealized, stationary density field for which we can compute the isoneutral slopes exactly, assuming that here the neutral surfaces align with the isopycnals. We do not consider any actual fluid dynamical setup, meaning there is no background flow $\left(\bar{u}=0\right)$. This three‐dimensional idealized test case is an extension of the two‐dimensional test case from Shah et al. (2011), and is given by

(22) $$\rho \left(x,y,z\right)=\rho_{0}\left[1-\frac{N^{2}z}{g}+A_{x}\;\sin\;\left(k_{x}x\right)+A_{y}\;\sin\;\left(k_{y}y\right)\right],$$

with *ρ*_0_ a reference density, *N* the Brunt‐Vaisala frequency, *g* the gravitational acceleration, *A* the amplitude of the wave‐like neutral surfaces, and *k* their wavenumber (subscripts denoting direction). The *z*‐coordinate of the neutral surface corresponding to the density ρ* is then found as

(23) $$z_{\text{iso}}\left(\rho^{*},x,y\right)=\frac{g}{N^{2}}\left[1-\frac{\rho^{*}}{\rho_{0}}+A_{x}\;\sin\left(k_{x}x\right)+A_{y}\;\sin\left(k_{y}y\right)\right].$$

We use a similar choice of parameters as (Shah et al., 2011), which is representative of the large‐scale ocean:

(24) $$\begin{array}{ccc} \rho_{0} & = & 1025\;kg\;\;m^{-3},\;N^{2}=1\times 10^{-5}s^{-2},\;g=10\;m\;\;s^{-2},\\ A_{x} & = & 1\times 10^{-3},\;A_{y}=1.1\times 10^{-3},\;k_{x}=k_{y}=\frac{2}{\pi }\times 1\times 10^{-5}m^{-1}. \end{array}$$

This choice of parameters leads to a maximum slope of max (|*S*|) ≈ 10^−3^, which is a typical value for neutral slopes in the ocean, and for which the small‐slope approximation (Equation (7a), (7b)) is valid (Mathieu & Deleersnijder, 1998). Although we may not use this approximation in the Markov‐1 model due to singularity, as explained in Section 2.3, it is useful to compare the small‐slope approximation of Markov‐0 (Equation (7a), (7b)) with its full formulation (Equation (7a), (7b)).

### 3.3 Spurious Diffusivity

We can compare the spurious dianeutral diffusivities induced by numerical errors in the discretized Markov‐0 and Markov‐1 models. We limit this analysis for brevity and refer the reader to Shah et al. (2011) for an extensive discussion of numerical errors introduced by Markov‐0. The models considered here have an equivalent effective diffusivity (Equation (7a), (7b)) in the limit *t* ≫ *T*_*L*_. We initialize 12,800 particles on a neutral surface, using a regular *xy*‐grid, with the *z*‐coordinates computed from Equation (7a), (7b) and ρ* = 1,027.5 kg m^−3^. We found that results are insensitive to adding more particles. We take into account the periodic topology of the neutral surfaces to make sure crests and troughs are sampled evenly. Then, we numerically integrate the particles for 90 days using several choices of integration timestep Δ*t*. The particle displacements are computed by using the exact density field (22) and its spatial derivatives. From the vertical departure of the particles from the neutral surfaces, we can compute an effective spurious vertical diffusivity,

(25) $$\kappa_{z,spurious}=\frac{{\left(\langle z-z_{\text{iso}}\rangle \right)}^{2}}{2T_{\text{int}}},$$

where the angled brackets denote a particle ensemble average and *T*_int_ is the total integration time. We use this as an approximation of the spurious dianeutral diffusivity introduced by the numerical approximation of Equation (7a), (7b).

In the Markov‐0 model, we set *κ* = 1,000 m^2^ s^−1^ and *ϵ* = 0, such that the only dianeutral movement of particles is due to numerical errors. We test both **K**_Redi_ and **K**_Redi, approx_. We cannot test Markov‐0 using **K**_LS_, as we do not consider a fluid setup with flow from which its parameters are computed.

For Markov‐1, we use a value of *T*_*L*_ = 20 days, and we determine *ν* ^2^ = *κ*/*T*_*L*_ = 5.79 × 10^−4^ m^2^ s^−2^, so that the effective isoneutral diffusivity in the diffusive limit equals the one used for Markov‐0 (see Equation (7a), (7b)). We also need to specify the nonzero dianeutral fading‐memory time and velocity variance in the Markov‐1 model to guarantee that Equation (7a), (7b) and Equation (7a), (7b) are nonsingular. To ensure rapid decorrelation of **u′** in the local dianeutral direction, we set $\varepsilon =\frac{\Delta t}{T_{L}}$ (Equation (7a), (7b)). In order to avoid ***θ*** being singular, we also need a nonzero *η*. However, here we are interested in the dianeutral movement induced by numerical errors, rather than what is specified by the algorithm. Here we need to make a trade‐off: we found that if *η* gets very small (*η* ≲ 10^−10^), this causes instabilities due to the multiplication of very small and very large terms (inverses of *η*) when computing the drift correction term (Equation (7a), (7b)). This may not necessarily lead to a spurious diapycnal diffusivity, but we found that it can lead to particle accumulation is specific areas. We choose *η* = 10^−8^; a value for which we do not observe noticeable instabilities with the drift correction term. For small choices of *dt*, this choice of *η* will cause the “spurious” diapycnal diffusivity to equal the expected diapycnal diffusivity (computed using Equation (7a), (7b)), while for larger timesteps the spurious diffusivity is dominated by numerical errors.

**Figure 1:**
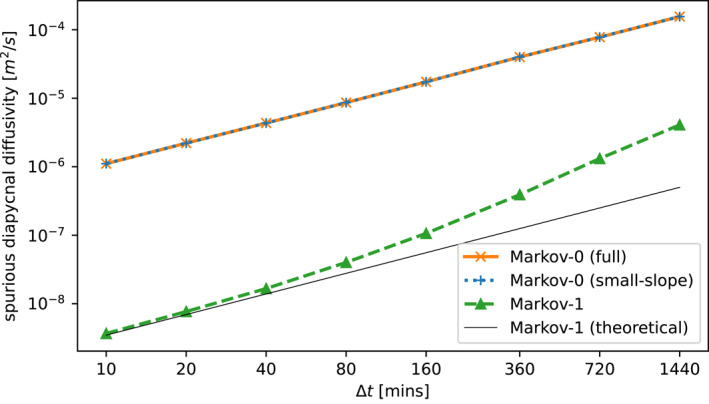
Spurious dianeutral diffusivities after 90 days in the Markov‐0 model (with and without the small‐slope approximation (Equation (7a), (7b))), and the Markov‐1 model, using several timesteps Δ*t*. For Markov‐1, we also plot the diapycnal diffusivity that is theoretically imposed through our choice of *η*. The Markov‐1 model has a much smaller spurious dianeutral flux for each timestep. Using the small‐slope approximation for Markov‐0 leads to negligible differences in the spurious diapycnal diffusivity.

Figure 1 shows that the spurious dianeutral diffusivity after 90 days of integration is much smaller for Markov‐1 than for Markov‐0. Recall that both use the same Euler‐Maruyama discretization scheme (Equation (7a), (7b)). The difference in dianeutral diffusivity is due to the fact that the expected turbulent displacement for a single timestep in Markov‐1 is *E* (‖**u′**‖ Δ*t*) = *ν*Δ*t* (see Equation (7a), (7b)), while that in Markov‐0 is $E\left(V\cdot dW\right)=\sqrt{2\kappa \Delta t}$, (see Equation (7a), (7b)) where *E* denotes the expected value and ‖ ⋅ ‖ the vector norm. The turbulent excursion of Markov‐1 in one timestep is therefore much smaller than that of Markov‐0 over the range of Δ*t* investigated here, and thus Markov‐1 introduces less dianeutral movement as the neutral surfaces curve. Also note that over this range of Δ*t* and with our choice of *κ*, *ɛ* and *η*, as *dt* increases, the diapycnal diffusivity diverges from the theoretical diapycnal diffusivity imposed through *η*. This divergence is caused by numerical errors, meaning these start dominating for the larger values in our range of *dt*. We conclude that Markov‐1 generally performs significantly better in keeping particles on idealized neutral surfaces. Note that the spurious diapycnal diffusivity depends on the slopes of the idealized neutral surfaces, determined by *A*_*x*_, *A*_*y*_, *k*_*x*_, and *k*_*y*_ (Shah et al., 2011).

Several studies propose the use of higher order numerical schemes to reduce the spurious dianeutral flux resulting from numerical integration (Gräwe, 2011; Gräwe et al., 2012; Shah et al., 2011) or the use of adaptive time‐stepping methods (Shah et al., 2013). While higher order schemes, such as the first order Milstein scheme (see Kloeden & Platen, 1999), indeed perform better in the idealized configuration, we find that this improvement is negligible when applied to discrete ocean model data using commonly used spatial and temporal output resolutions (see Section 4.1), and a Lagrangian timestep of 40 min, indicating that the error introduced by interpolating Eulerian data dominates that of the numerical method.

### 3.4 Well‐Mixedness

The equations for the Markov‐1 model, including the drift‐correction term (Equation (7a), (7b)), are rigorously derived in Berloff and McWilliams (2002). However, since we create an ad‐hoc adaption of this model for use in three‐dimensional isoneutral situations, it is important that we verify whether we did not inadvertently violate the well‐mixed condition. Rather than rigorously proving the WMC, we take a pragmatic approach here and visually inspect particle distributions to see if we can find spurious accumulation. We choose pragmatism over rigor of proof, because in applications with discrete Eulerian ocean model output, Lagrangian simulations with Markov‐0 and Markov‐1 are both affected by numerical errors due to discretization and interpolation. These numerical aspects will violate the WMC in any case, hence a pragmatic visual verification of the WMC satisfies our needs.

**Figure 2:**
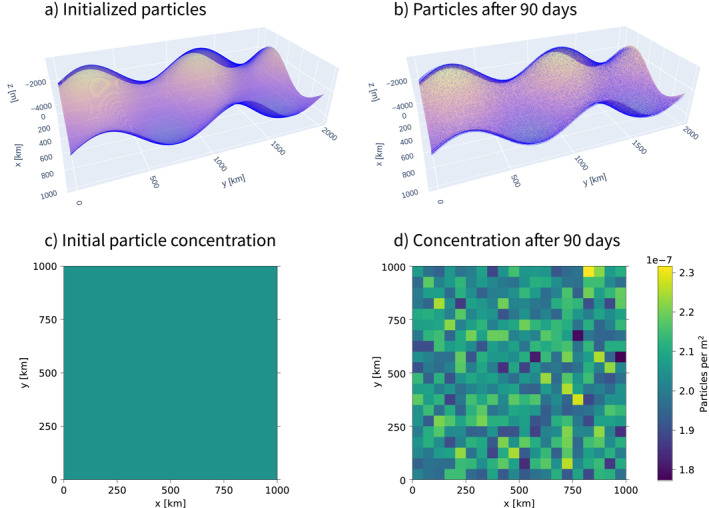
(a) 204,800 particles on an idealized neutral surface, initialized in a regular *xy*‐grid. (b) the same particles after 90 days of integration with the Markov‐1 model, with *T*_*L*_ = 20 days and *ν* ^2^ = 5.79 × 10^−4^ m^2^ s^−2^. (c) initial concentration computed by binning particles in (a) and dividing by the total area of curved surface per bin. We take advantage of the periodicity of the domain and analyze all particles over one wavelength 1/*k*_*x*_ = 1/*k*_*y*_ = 1,000 km by displacing them as *x* = *x* mod 1/*k*_*x*_, *y* = *y* mod 1/*k*_*y*_. (d) final particle concentration after 90 days of integration. Concentrations in (d) are much less homogeneous than they are initially in (c), but there are no clear accumulation patterns coinciding with specific features of the idealized neutral surface.

To visually inspect any spurious particle accumulation, which would indicate a WMC‐violation, we integrate 204,800 particles with the Markov‐0 and Markov‐1 models for 90 days and investigate particle distributions. Figures 2a and 2b shows the initial and final particle distributions on our idealized neutral surfaces for Markov‐1. We again set *T*_*L*_ = 20 days and *ν* ^2^ = *κ*/*T*_*L*_ ≈ 5.79 × 10^−4^ m^2 ^ s^−2^, so that the effective diffusivity after 90 days (in the diffusive limit) is approximately *κ* ≈ 1 × 10^3^ m^2^ s^−1^. Figures 2c and 2d shows the initial and final particle concentrations in the *xy*‐plane, obtained by binning particles and dividing by the area of curved neutral surface per bin. We do not observe any distinct zones in which particles accumulate. Since the input to the Markov‐1 model in this test case solely consists of the ***σ*** and ***θ*** tensors, whose elements in turn depend on the slopes of the neutral surfaces, any spurious accumulation should manifest itself at specific slope levels. Since we do not observe this, this indicates that in this stationary situation without background flow the WMC is not violated by our ad‐hoc isoneutral formulation of Markov‐1.

## 4 Dispersion in an Antarctic Circumpolar Current Channel Model

We also compare the Markov‐0 and Markov‐1 models through Lagrangian simulations using the output of an ocean model. We use two types of Eulerian model fields at a 50 km horizontal spacing: one is the output of an ocean model run at this coarse resolution, and the other is a coarsened output of a fine‐resolution 5 km model. The fine‐resolution data serves as an eddy‐resolving reference case. While the coarse‐resolution data is most representative of the coarse models for which Lagrangian subgrid‐scale models are useful, the coarsened data allows for easier comparison to the fine‐resolution reference case.

First, we look at how well Markov‐1 reproduces the specified Lagrangian integral timescale and effective diffusivity in the diffusive limit. Then, we qualitatively compare particle trajectories produced by Markov‐0 and Markov‐1 with those produced by advection only. We also compare the spread of a patch of Lagrangian particles, in analogy to a tracer patch experiment. Finally, we estimate the spurious dianeutral diffusivities introduced by the different models.

In each experiment, we use single values for the isoneutral Lagrangian integral time and isoneutral velocity variance. This means that we assume a homogeneous and stationary situation without boundary effects. The stationarity assumption is valid for the coarsened and coarse fields, but the other assumptions are not. To deal with inhomogeneity, we could use space‐dependent and anisotropic tensors for ***σ*** and ***θ***, but since future applications are likely to use constant parameters, we choose the pragmatic route and do so as well.

Since we use Eulerian data with boundaries, we need to consider boundary conditions. In a two‐dimensional stationary and homogeneous setting, perfect reflection satisfies the WMC (Wilson & Flesch, 1993). Although neutral surfaces in the Southern Ocean can outcrop at the surface (Marshall & Speer, 2012), we use the assumption that neutral slopes at the lateral boundaries are near‐flat, and adopt perfect reflection as our choice as well. The isoneutral slopes in certain areas of the model data may be unrealistically large due to spurious effects, so we use a tapering scheme based on that of Danabasoglu and McWilliams (1995) to lower or turn off turbulent displacements in such regions. Details of the tapering mechanism are found in Appendix A.

### 4.1 Eulerian Model Description

We use a simplified model of the Antarctic Circumpolar Current run in MITgcm (Campin et al., 2020; Marshall et al., 1997), similar to the channel model used by Abernathey et al. (2011) and Balwada et al. (2018). We use an adaptation that is extensively described in MITgcm's documentation, also available at: https://mitgcm.readthedocs.io/en/latest/examples/reentrant_channel/reentrant_channel.html. It consists of a zonally re‐entrant channel that is 1,000 km long in the zonal (*x*) direction, 2,000 km wide in the meridional (*y*) direction, and 3,980 m deep. The model consists of 49 vertical levels that range from 5.5 m depth at the surface to 149 m at depth. It is forced by a constant sinusoidal wind stress and a temperature relaxation at the surface and northern boundary. The equation of state is set linearly dependent to potential temperature only, causing the neutral surfaces to coincide with surfaces of constant potential temperature. This allows us to compute neutral slopes using Equation (7a), (7b). To break zonal symmetry, a meridional, Gaussian‐shaped ridge is placed in the center of the domain, going up to 2382.3 m *m* depth. The ridge has a small opening in the center, causing a strong barotropic jet to develop.

**Figure 3:**
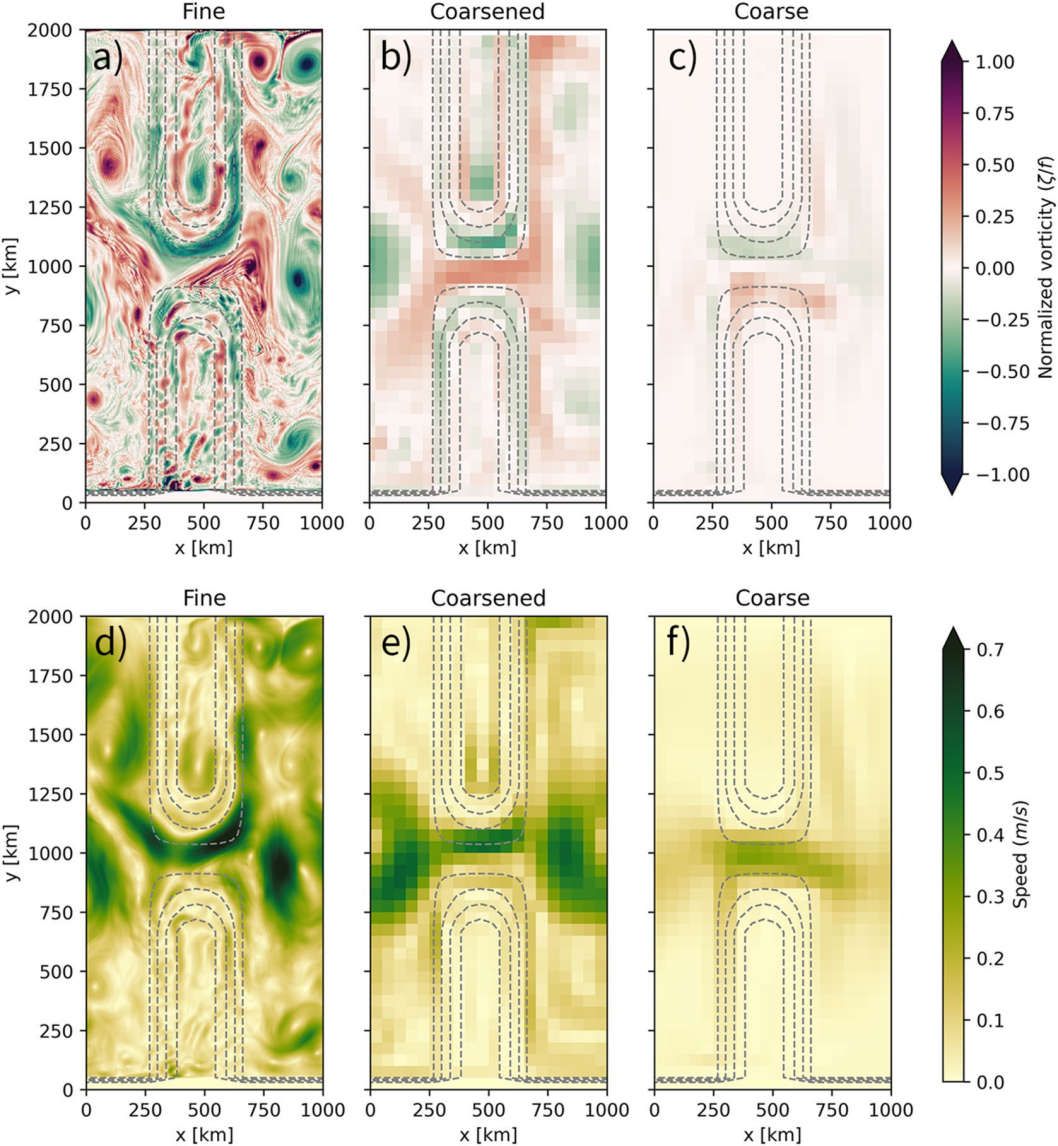
Snapshot of the vorticity (a–c) and speed (d–f) of the fine (a and d), coarsened (b and e), and coarse (c and f) model fields used in this study. The fine fields are daily averages, the coarsened fields are 1‐year time averages and 50 km spatial averages, and the coarse model is in steady state. Dashed lines indicate the position of the meridional ridge.

The model is spun up for 100 years and run at two horizontal resolutions: once at 5 km resolution (fine‐resolution), at which the mesoscale eddies are resolved, and once at 50 km resolution (coarse‐resolution) where eddies cannot develop. Daily averages of the output data are used for the Lagrangian simulations. The coarse‐resolution flow is in steady‐state, exhibiting no temporal variability. We also create a coarsening of the fine‐resolution model in space and time, by taking a yearly time‐average of the flow and spatially averaging velocities and temperature fields over 50 km. These coarsened fields thus include the effect of eddies on the mean flow. Snapshots and means of the vorticity and speed fields in the fine, coarsened and coarse runs are found in Figure 3. The derivatives of the density field, used for computing the neutral slopes, are computed by means of grid‐aware central differences using the XGCM package (Abernathey et al., 2021).

### 4.2 Parameter Estimation

To use the two Markov models in our experiments, we need to identify *κ* for Markov‐0 (except when using the LS parameterization) and *T*_*L*_ and *ν* ^2^ for Markov‐1. We can estimate globally representative values from Lagrangian quantities of the fine‐resolution flow field. To do so, we first compute Lagrangian particle trajectories with the fine‐resolution model output. We initialize 64,860 Lagrangian particles released regularly spaced apart 20 km in the horizontal and 200 m in the vertical, with −200 *m* ≥ *z* ≥ −1,600 m in order to stay away from the mixed layer and the ridge. We then integrate the trajectories using a fourth order Runge‐Kutta scheme, with a timestep Δ*t* = 40 min for 180 days.

**Figure 4:**
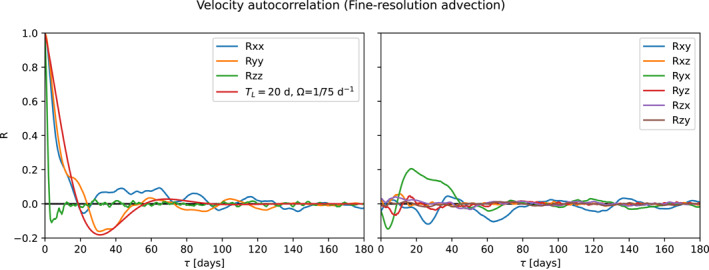
Lagrangian autocorrelations in the fine‐resolution model, including an exponentially decaying and oscillatory function (Equation (7a), (7b)) with *T*_*L*_ = 20 days and *Ω* = 75 days.

The Lagrangian integral time is related to the Lagrangian autocorrelation (Equation (7a), (7b)). Figure 4 shows the Lagrangian autocorrelation estimated from particle trajectories in the fine‐resolution model. We can clearly see the oscillatory and exponentially decaying behavior of the horizontal autocorrelations. Similar to Sallée et al. (2008), we approximate the Lagrangian autocorrelation to be decomposable as

(26) $$R\left(\tau \right)=\cos\left(2\pi \Omega \right)\;e^{-\tau /T_{L}},$$

where Ω is the frequency of the oscillation. While the parameters *T*_*L*_ and Ω can be estimated using a least‐square fit, we are only interested in approximate values for the parameters. A choice of *Ω* = 1/75 per day and *T*_*L*_ = 20 days approximates the autocorrelation functions well enough for our purposes. Bear in mind, though, that we only continue with *T*_*L*_, as Markov‐1 cannot reproduce the oscillatory behavior of particle dispersion in the ocean.

Having fixed *T*_*L*_, we only need to estimate *κ*, since this will readily give us an average value of *ν* ^2^ that reproduces the correct diffusivity in the dispersive regime through (14; Koszalka et al., 2013). The absolute diffusivity tensor (LaCasce, 2008) is found by integrating the Lagrangian autocovariance:

(27) $$K_{ij}\left(x,\tau \right)=\int_{0}^{\tau }\langle u_{i}^{\prime }\left(t_{0}|x,t_{0}\right)u_{j}^{\prime }\left(t_{0}+\tilde{\tau }|x,t_{0}\right)\rangle d\tilde{\tau }.$$

To find the isoneutral diffusivities, *i* and *j* should coincide with the principal directions of the neutral plane at each location. However, since the isoneutral slope in our model is small (generally of order 10^−3^), we will estimate the isoneutral diffusivity from *K*_xx_ and *K*_yy_.

**Figure 5:**
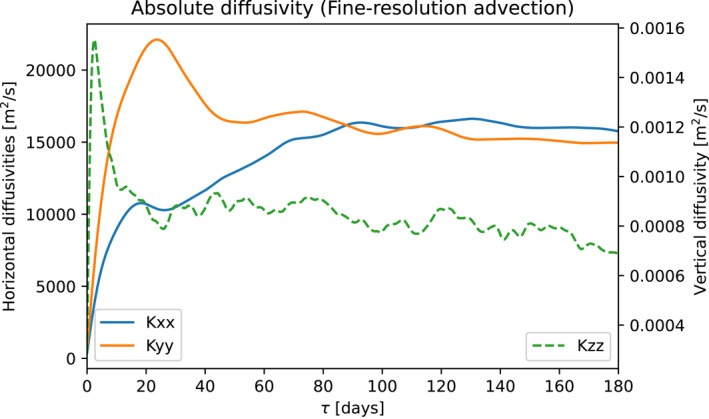
Absolute diffusivities *K*_xx_, *K*_yy_, and *K*_zz_, in the fine‐resolution model, computed through (27).

Figure 5 shows the horizontal and vertical absolute diffusivities over time. The absolute diffusivity corresponding to the diffusive limit, in which Markov‐0 is valid, is found at *τ* ≫ *T*_*L*_, for which the diffusivity should take on a near‐constant value. Theoretically, it is found by integrating Equation (7a), (7b) to infinity, but in practice, it can be found by integrating past the negative and positive lobes associated with the oscillatory component of the Lagrangian autocorrelation, when the diffusivity becomes near‐constant (Griesel et al., 2014; Klocker, Ferrari, Lacasce, & Merrifield, 2012). From Figure 5, we estimate the isoneutral diffusivity to be similar to the horizontal absolute diffusivity, with a value of *κ* = 1.5 × 10^4^ m^2^ s^−1^.

### 4.3 Lagrangian Integral Time and Diffusivity From the Markov‐1 Model

Now we initialize particles in the same lattice as used in Section 4.2 and apply the Markov‐1 parameterization. We simulate trajectories by integrating the stochastic differential Equation (7a), (7b) using the Euler‐Maruyama scheme (Equation (7a), (7b)) for 180 days, with Δ*t* = 40 min. We set *T*_*L*_ = 20 days, and specify *ν* ^2^ = 8.68 × 10^−3^ m^2 ^ s^−2^ in order to obtain an effective diffusivity of 1.5 × 10^4^ m^2^  s^−1^ in the diffusive limit. We also set *η* and *ɛ* in such a way that the effective dianeutral diffusivity in the limit *t* ≫ *T*_*L*_ is 1 × 10^−5^ m^2 ^ s^−1^. These settings are used in the remainder of this study. Derivatives of Eulerian quantities that are necessary for computing the tensor elements of ***σ*** and ***θ*** (and later **K**) are computed with central differences and successively interpolated linearly in space. Our aim is to see how well the model reproduces the diffusivity and Lagrangian timescale that we specified, to verify our ad‐hoc dianeutral formulation of Markov‐1.

**Figure 6:**
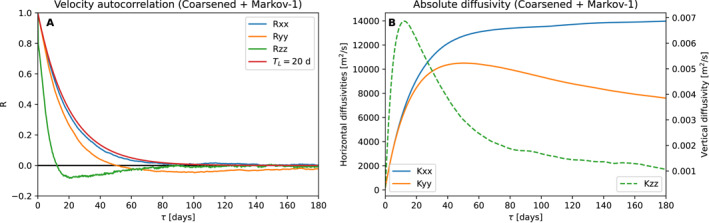
Lagrangian autocorrelation and absolute diffusivity produced by the Markov‐1 model when applied on the coarsened field. The Lagrangian autocorrelation in the *x*‐direction best resembles that of an exponentially decaying function with a 20‐day *e*‐folding timescale (in red for reference).

**Figure 7:**
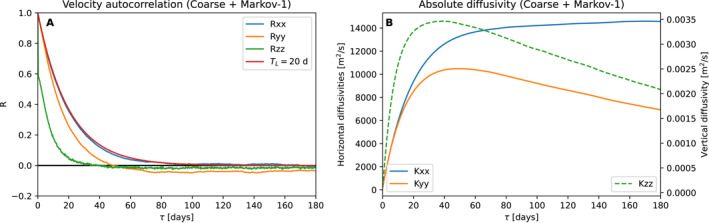
Lagrangian autocorrelation and absolute diffusivity produced by the Markov‐1 model when applied on the coarse field (cf. Figure 5). The Lagrangian autocorrelation in the *x*‐direction best resembles that of an exponentially decaying function with a 20‐day *e*‐folding timescale (in red for reference).

Figure 6 shows the Lagrangian autocorrelation and absolute diffusivity of particles simulated using the Markov‐1 subgrid‐scale model using the coarsened field, similar to Figures 4 and 5. Figure 7 provides a similar diagram for the coarse field. The exponential decay with an *e*‐folding timescale of 20 days can be clearly seen in the autocorrelation. There is a clear absence of the oscillatory component, which Markov‐1 is unable to simulate.

The absolute diffusivity of 1.5 × 10^4^ m^2 ^s^−1^ is not fully reproduced. In the *x*‐direction, values reach up to approximately 1.4 × 10^4^ m^2^ s^−1^, but in the *y*‐direction, they are much smaller, with a maximum of 1.0 × 10^4^ m^2^ s^−1^ and a decrease at larger time lags. There are two reasons why values do not reach 1.5 × 10^4^ m^2^ s^−1^. First, in regions where the slope is unrealistically high, for example, in the direct vicinity of the meridional ridge, turbulent velocities are tapered to zero (see Appendix A), which decreases the absolute diffusivity computed from the particle ensemble. Second, the lateral domain boundaries limit the dispersion of material and therefore also cause a decrease in diffusivity, as *D*_yy_ cannot grow linearly over long timescales. While the effect of tapering likely plays a role for both *K*_*xx*_ and *K*_yy_, only *K*_yy_ is affected by boundaries, which causes it to decrease over time. We clearly see that *R*_zz_ has a much shorter *e*‐folding time than 20 days. This is likely due to the effect of curvature in the neutral surfaces, and the rapid decorrelation we impose in the dianeutral directions (Equation (7a), (7b)).

### 4.4 Individual Trajectories

A typical aim of Lagrangian subgrid‐scale dispersion models is to construct realistic synthetic particle pathways in the absence of turbulent eddies. It is therefore illustrative to plot particle trajectories generated by advection using the three model fields (fine, coarsened and coarse) and compare those with trajectories generated by Markov‐0 and Markov‐1. To do so, we randomly subsample 100 trajectories that were initialized on the same lattice as used in Section 4.2. We again use the Runge‐Kutta 4 scheme for advection and Euler‐Maruyama for the Markov models, a timestep Δ*t* = 40 min, and a simulation time of 180 days. Like in the previous section, we tuned Markov‐1 to produce a diapycnal diffusivity of 1 × 10^−5^ m s^−2^, and now we do the same for Markov‐0 by setting *ϵκ* accordingly. These parameters will also be used for the remainder of this paper. To more easily identify re‐entering trajectories, we record when particles cross the periodic boundary, so that we can plot particle trajectories as unbroken paths by repeating the periodic domain in the zonal direction.

**Figure 8:**
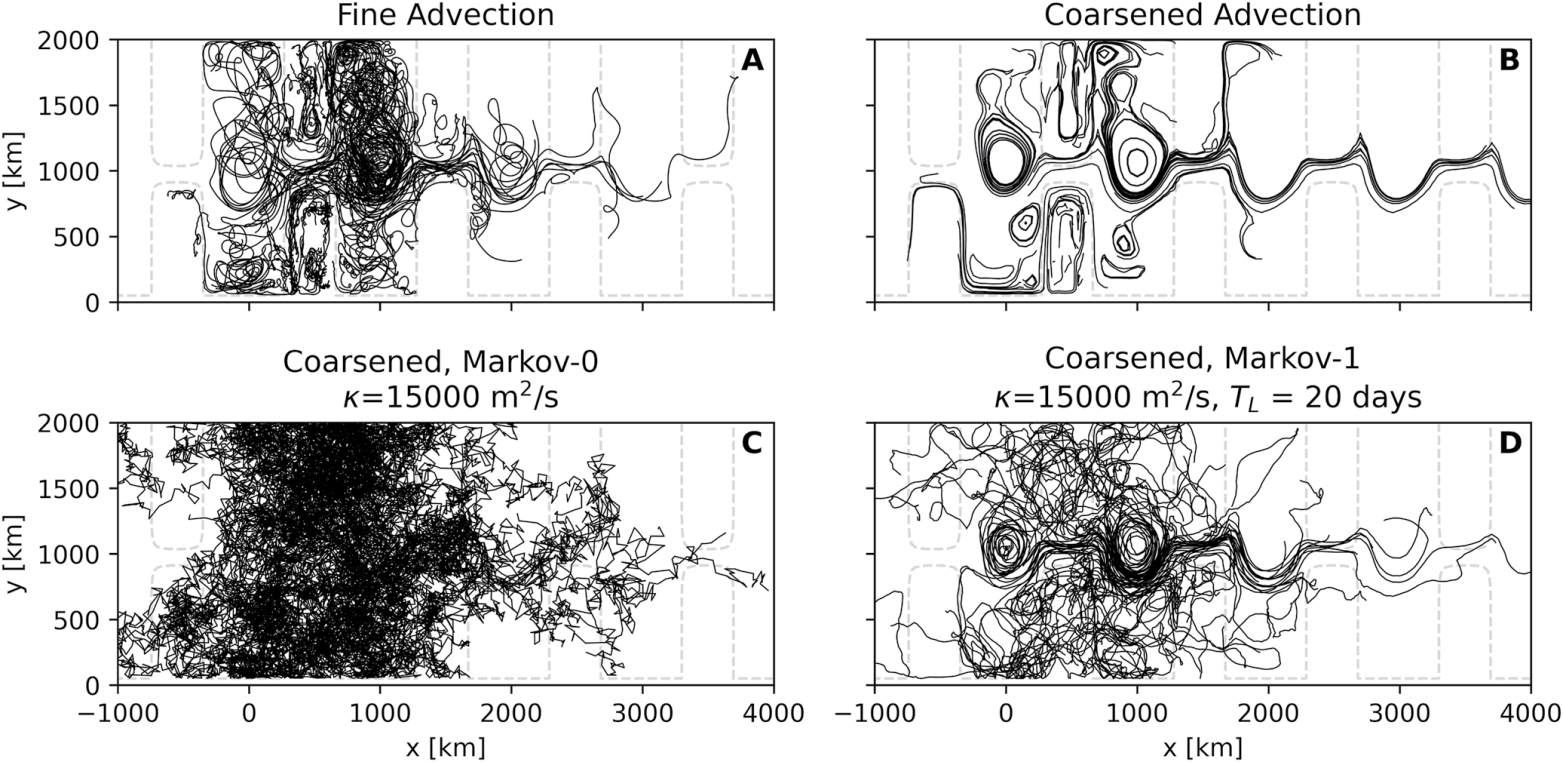
100 randomly subsampled trajectories from 180 days of simulation on (a) fine‐resolution and (b) coarsened fields, and using coarsened fields in combination with (c) the diffusive parameterization and (d) Markov‐1. While the domain is periodic, here we tile it in the zonal direction, to separate particles crossing the zonal periodic boundaries. The −3900m isobath is plotted with dashed gray lines, indicating the location of the ridge in the periodic channel.

Figure 8 considers 100 trajectories from Markov‐0 and Markov‐1 in the coarsened case, compared to advection using fine‐resolution and coarsened fields, which serve as reference. These trajectories are released at different horizontal and vertical locations, subsampled from the lattice used in the previous two sections. From the trajectories in Markov‐0 we clearly see that there is no autocorrelation in the particle velocities, with the directions in which a particle moves rapidly changing between recorded timesteps. Particles simulated with Markov‐0 also travel much more, as the turbulent displacement in this model is much larger than that of Markov‐1 (see the discussion in Section 3.3). Markov‐1 clearly does a better job at simulating the trajectories from the fine‐resolution reference run. A major difference is that trajectories in the fine run exhibit looping motions. While the trajectories in Markov‐1 veer over time, it is unable to produce the looping motions that are seen in the fine‐resolution run (Veneziani et al., 2005). Bear in mind that in the stochastic perturbations between different particles advected by Markov‐0 and Markov‐1 are uncorrelated. Instead, each particle “feels” its own turbulent field.

**Figure 9:**
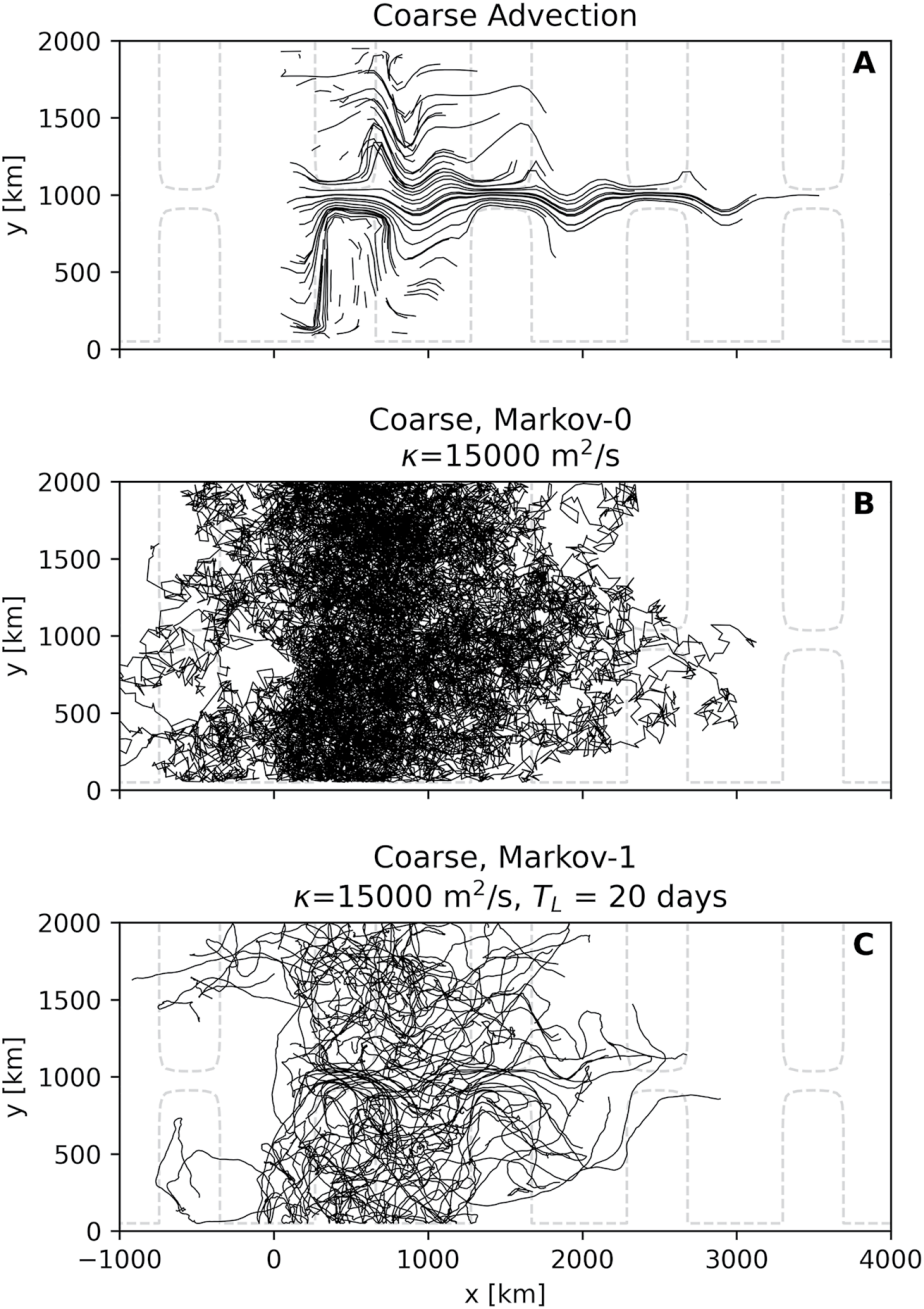
Same as Figure 8, but using coarse‐resolution fields.

Figure 9 considers the coarse‐resolution case. In this case, the underlying flow field has no eddies. When comparing trajectories produced by the Markov models, we thus have no eddying reference case. In the advection‐only case, the absence of strong dispersion is clear. One major difference with the results from the coarsened case is the absence of any stationary meanders. Trajectories produced by Markov‐1 again seem the most realistic when compared to Figure 8a, albeit less obviously than was the case for Figure 8d.

### 4.5 Tracer Spread

In analogy to studying the spread of a small patch of tracer (Wagner et al., 2019), we qualitatively compare the spread of a patch of Lagrangian particles advected in the fine‐resolution, coarsened, and coarse‐resolution fields and apply the Markov‐0 and Markov‐1 subgrid‐scale models to the later two flows. For Markov‐0, we use the isotropic isoneutral diffusion tensor **K**_Redi, approx_ (Equation (7a), (7b)) and the LS parameterization **K**_LS_ (Equation (7a), (7b)). For the LS parameterization, we set *C* = 1.

**Figure 10:**
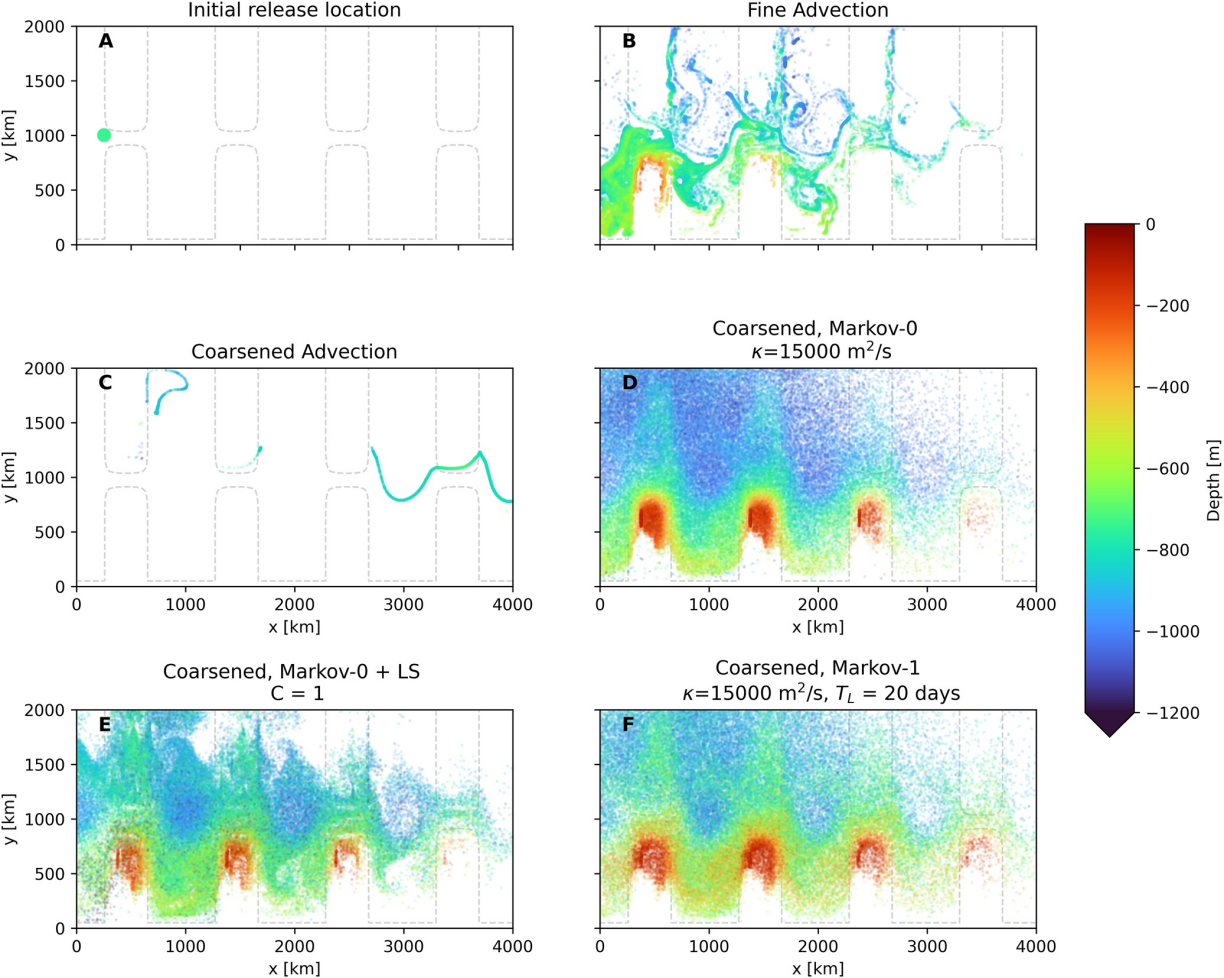
(a) Initial particle positions at *z* = −736 m, (b–f) show particle locations and depths after 180 days of simulation with Δ*t* = 40 min (b and c) show particles advected with the fine‐resolution and coarsened model fields, while (d–f) use the diffusion/Markov‐0 and Markov‐1 models. Particles that fall within the mixed layer are not shown (see Appendix B).

We initialize a patch of particles initially located at *z* = −736 m (corresponding to the 25th vertical level) in a radius of 50 km centered around (*x* = 250 km, *y* = 1,000 km), see Figure 10a.

Figure 10 shows the particle distributions after 180 days of simulation, using advection and the different subgrid‐scale models on the coarsened flow data. Again, we repeat the domain in the zonal direction, so that we can distinguish particles that have crossed the periodic boundary. Figure 10c shows the obvious need for modeling subgrid‐scale dispersion when turbulent flow features are filtered out.

Figures 10d–10f show similar patterns when compared to one another, albeit with the dispersion in the LS case being somewhat weaker, and particles in the Markov‐0 case reaching deeper than the others. Note that the diffusivity in the LS parameterization is solely determined by derivatives of the flow fields. The pattern in 10e is qualitatively similar to 10b, which bears testimony to the skill of the LS parameterization. Since the particles in the parameterizations each experience their own independent turbulent fields, coherent structures and filamentation as seen in 10b cannot be reproduced by the Markov models.

In both Markov‐0 models and in the Markov‐1 model, we see some spurious particle accumulation on the left side of the ridges (at *x* = 500 km + *n* ∗ 1,000 km, with *n* = 0, 1, 2, … ). In the LS case, these accumulation patterns (or patterns where particles are fully absent) occur at other places too. In all cases this is likely due to sharp changes in the discrete derivatives used for computing the slopes that are necessary for filling the elements of **K**, ***σ***, and ***θ***. The LS parameterization relies on discrete derivatives of more quantities for computing its tensor elements, since these also depend on the shear of the flow (see Equation 6). It is therefore more susceptible to violations of the WMC when these discrete derivatives change strongly in space and interpolation is used.

**Figure 11:**
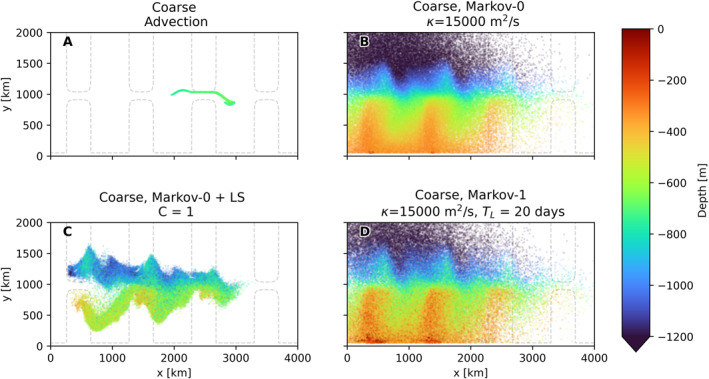
Like 10, with (a) advection in coarse‐resolution model, (b–d) using the different subgrid‐scale models.

Figure 11 shows the spreading of Lagrangian particles in the coarse model. Again, the isotropic Markov‐0 model and Markov‐1 show a similar spread of particles, with particles in Markov‐0 again reaching slightly larger depths. However, the LS parameterization this time produces very different results, with the dispersion being much more limited, and the particles being more concentrated. This means that in this case the shear‐based parameterization leads to much smaller diffusivities in **K**_LS_. This makes sense, as the fine‐resolution flow field (and thus the coarsened flow) is full of baroclinic instabilities that lead to eddies with large shear. The resolution in the coarse model is too low for these instabilities to develop. Instead, the flow tends to a much smoother steady‐state, with less shear. As this yields smoother derivatives in the temperature field (and in the velocity fields in the case of LS), this should lead to less spurious accumulation. Indeed we see no clear regions where particles accumulate.

### 4.6 Spurious Dianeutral Diffusivity

Two possible causes of spurious dianeutral tracer fluxes are numerical integration and interpolation of discrete, time‐evolving Eulerian flow fields. The spurious dianeutral flux can be expressed as a diffusivity, and this diffusivity should be as small as possible compared to the vertical diffusivity that is specified to represent dianeutral processes. For example, in the Southern Ocean, the average diapycnal diffusivity at 1,500 m depth is estimated to be 1.3 ± 0.2 × 10^−5^ m^2^ s^−1^ (Ledwell et al., 2011). It is important to assess how large the dianeutral diffusivities in our Lagrangian simulations become, and how they compare to the dianeutral diffusivity that we specify. In this section, we will assess these spurious dianeutral diffusivities. In these experiments, we specified an explicit dianeutral diffusivity of 1 × 10^−5^ m^2^ s^−1^. Moreover, in the case of the Markov models, we test several values of (effective) isoneutral diffusivities, keeping *T*_*L*_ = 20 days in the case of Markov‐1. For Markov‐0 combined with the LS parameterization, we choose different tuning parameters *C* at O(1), which affect the strength of the diffusivity.

We compute the effective dianeutral diffusivity in the case of pure advection using the fine‐resolution, coarsened, and coarse‐resolution fields, and using the Markov‐0 and Markov‐1 model. This dianeutral diffusivity is approximated as follows: for each particle, we record its initial local water density. Then, after simulating the particle's movement for 180 days, at the particle's new horizontal location, we compute the depth *z*_iso_ of the neutral surface corresponding to the original local water density. Comparing this depth with the particle's new depth, we can compute a spurious vertical diffusivity (similar to Equation 25). This again assumes that the dianeutral diffusivity is closely aligned with the vertical direction. We separate the results for three depth classes on which particles were released. Particle trajectories that at any point reach depths of −50 m or higher are excluded in these computations, in order to filter out effects related to particles entering the mixed layer (see Appendix B).

The results are found in Table 1 for the coarsened flow and in Table 2 for the coarse‐resolution flow. In all cases, the effective dianeutral diffusivities are larger than the value of 1 × 10^−5^ m^2^ s^−1^ that we explicitly set, meaning that the spurious dianeutral diffusivities due to errors in interpolation and the numerical schemes dominate. This is already the case for simulations that only use advection. We found that halving the timestep does not make a difference here, indicating that the error in the case of advection is likely not due to the time discretization. In the case of advection using fine‐resolution data, the distance that a particle covers over the course of one flow snapshot, compared to the length of a grid cell, is relatively larger than in the case of coarse‐resolution data, where it takes longer to traverse the larger cells. The dianeutral error can then be reduced by using more frequent snapshots of the data (e.g., 6‐hourly snapshots instead of daily), such that temporal interpolation occurs over a smaller time window (Qin et al., 2014). This could however come at a large expense in storage, memory and I/O. Here, we are solely interested in comparing the errors between different Lagrangian simulations, so we accept that the dianeutral diffusivities are larger than specified. In the coarsened and coarse‐resolution fields, we use steady‐state flows, meaning that the errors are due to spatial interpolation of coarse data, with time‐interpolation playing no role.

**Table 1:**
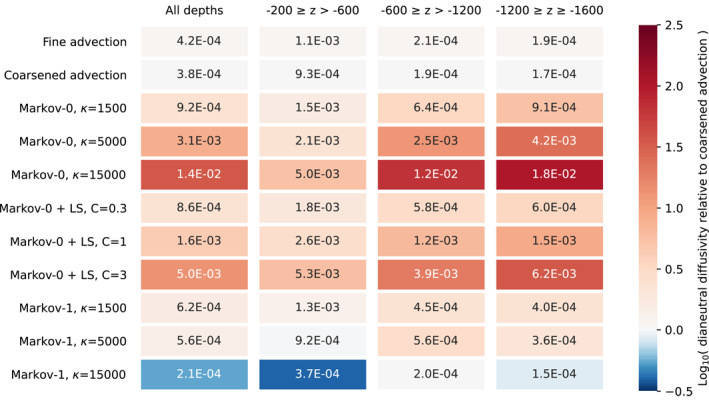
Effective Dianeutral Diffusivity (in m^2^ s^−1^) for Different Depth Classes With Parameterizations Applied on the Coarsened Flow Field, After Numerical Integration for 180 Days, With Δ*t* = 40 Minutes

*Note*. The color scale indicates the logarithm of the relative dianeutral diffusivity, when divided by the dianeutral diffusivity in the coarsened case per depth class as reference. This indicates the orders of magnitude that the dianeutral diffusivity differs from that in the simulations with only advection using coarsened fields. Of the parameterizations, Markov‐1 has the smallest dianeutral diffusivity, in some cases even smaller than in the simulation with advection only.

**Table 2:**
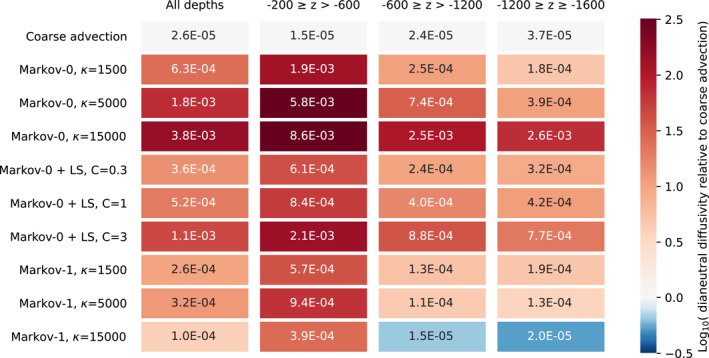
Same as Table 1, but Using Coarse‐Resolution Flow Fields

*Note*. Again, Markov‐1 has the Lowest Dianeutral Diffusivity of the Three Parameterizations.

Both Tables 1 and 2 show that for each experiment Markov‐0 produces a much larger spurious dianeutral diffusivity than Markov‐1. This corroborates the findings of Section 3.3. A likely explanation is that the isoneutral turbulent displacement in each of the models becomes somewhat dianeutral as discrete neutral surfaces “curve”, while the displacements in Markov‐1 are much smaller than is the case for Markov‐0. In the case of Markov‐0, we see the error increasing as the diffusivity increases. This pattern cannot be seen for Markov‐1, where in some cases, the error decreases with increasing effective diffusivity. Unfortunately, we do not have an explanation for this pattern.

Since the dianeutral diffusivity in the case of Markov‐0 can become several orders of magnitude larger than is the case for only advection, future studies should be careful with applying this subgrid‐scale dispersion parameterization. Here we implemented the Euler‐Maruyama scheme. Higher‐order schemes, such as the first order Milstein scheme, are able to greatly reduce the dianeutral error in idealized situations (Gräwe, 2011; Shah et al., 2011, 2013). However, we found that the Milstein‐1 scheme produces similar dianeutral errors to Euler‐Maruyama when applied on our coarsened and coarse‐resolution flows, further indicating that the cause of the error lies in interpolation combined with large turbulent displacements.

## 5 Conclusion

We achieved two main goals: formulating an isoneutral description of the Markov‐1 model, and extending an anisotropic tracer diffusion parameterization to the random walk dispersion/Markov‐0 model. With these goals, we aim to improve the parameterization of unresolved isoneutral turbulent motions due to eddies in Lagrangian studies.

Because of the inclusion of a velocity autocorrelation, the Markov‐1 model is able to produce both the ballistic and diffusive dispersion regime, and it produces particle trajectories and dispersion patterns that are more realistic than those produced by Markov‐0. Our formulation of Markov‐1, inspired by Redi's diffusion tensor, also has a much smaller spurious dianeutral flux than Markov‐0, due to the smaller turbulent displacement in each timestep. Large turbulent displacements in the isoneutral direction in the presence of curvature in the neutral surfaces lead to dianeutral excursions. Therefore, our three‐dimensional isoneutral formulation of Markov‐1 will hopefully be useful to the Lagrangian community, with the many benefits of higher order stochastic models beyond Markov‐1 given by previous studies (Berloff & McWilliams, 2002; Griffa, 1996; Veneziani et al., 2004). We also believe that the isoneutral formulation of the parameter tensors (Equation (7a), (7b) and Equation (7a), (7b)) is extendable to the parameter tensors of the higher order stochastic models beyond Markov‐1, as well as other improvements to this model, like the inclusion of looping motions.

Further research into the isoneutral formulation of Markov‐1, as well higher order stochastic models, may focus on better retaining the velocity autocorrelation on curved surfaces, which remains a complex issue (Gaspari & Cohn, 1999). Next to that, it may also further investigate boundary conditions further, as well as how Lagrangian particle models can transition from isoneutral dispersion in the ocean interior to horizontal and vertical mixing in the mixed layer, which has been left out of this study (see Appendix B). Moreover, future studies employing isoneutral dispersion models may benefit from improved computation of neutral surface slopes (Groeskamp et al., 2019).

We hope that future Lagrangian studies using coarse fields, such as the output of coupled Earth system models, may also benefit from the LS parameterization, as well as other Eulerian anisotropic parameterizations based on closure. This may help automatically determine the strength of the eddy diffusivity in different regions in the domain. When applied to the coarsened flow field, the LS parameterization was able to produce particle distributions similar to the isotropic Markov models, meaning that LS may obviate the need for explicit parameter estimation in Markov‐0. Our discussion of the LS parameterization may inspire further investigation into the application of closure schemes in Lagrangian simulations. Similarly, such closures could be further studied for the Markov‐1 model, although so far Berloff and McWilliams (2003) tested a related closure based on shear with negative results.

## Data Availability

Data and Software Availability Statement Lagrangian datasets (CC‐BY) and data generation and analysis scripts (MIT license) for this research are available at https://doi.org/10.24416/UU01-RXA2PB. This includes MITgcm model generation scripts and documentation, data post‐processing scripts, Parcels Lagrangian simulation scripts and analysis scripts for generating figures and tables.

## Appendix A Tapering Scheme

We use central differences to compute the neutral slopes *S*_*x*_ and *S*_*y*_ (Equation (7a), (7b)) in the discrete Eulerian model data in the experiments in Section 4. Incidentally, the computed neutral slopes can be unrealistically high, for example, in the vicinity of the meridional ridge or in the mixed layer (see Appendix B). The buoyancy in the mixed layer is mostly uniform, but if we compute neutral slopes in this region, small deviations in the local buoyancy field can lead to huge slopes. It is common practice in Eulerian ocean modeling to limit or turn off isopycnal/isoneutral diffusion in regions with high slopes, in order to prevent numerical instability. This practice is called “tapering”.

Here we use a tapering scheme similar to that of Danabasoglu and McWilliams (1995) to smoothly decrease the values of the Markov‐0 diffusivity tensors **K**_redi, approx_ and **K**_LS_ to zero in regions with high slopes. Similarly, for Markov‐1, we use it to smoothly decrease the perturbative velocity **u′** to zero in such regions. At each timestep, we respectively multiply **K**_redi, approx_, **K**_LS_, or *u′* by a taper function *f*_taper_ which assumes values between one in regions where the isoneutral slopes are well‐behaved and 0 in regions where it is unrealistically high. Danabasoglu and McWilliams (1995) choose a taper function

(A1)
$$f_{\text{taper,DMW}}(S)=\frac{1}{2}\left(1+\tanh\left[\frac{S_{c}‐\left|S\right|}{S_{d}}\right]\right),$$

where *S*_*c*_ is the slope at which *f*_taper_ = 0.5 and *S*_*d*_ an acting distance over which *f*_taper_ changes steeply. If we were to multiply the perturbative velocity **u′** in the Markov‐1 model (Equation (7a), (7b)) with such a function, this causes an exponential decay of the **u′** with an *e*‐folding timescale of Δ*t*/log (*f*(*S*)). This can significantly shorten the effective decorrelation of **u′** as set by *T*_*L*_. For example, in a simulation with *T*_*L*_ = 20 days and *dt* = 40 min, if *f*(*S*) persistently equals 0.999, this causes **u′** to exponentially decay with a timescale of 28 days. In conjunction with the exponential decorrelation specified using *T*_*L*_, this leads to an effective decorrelation with an *e*‐folding timescale of 12 days. This is why we limit the slope values over which tapering happens smoothly to values that differ from *S*_*c*_ by at most 3*S*_*d*_. We thus use the following taper function

(A2)
$$f_{\text{taper}}\left(S\right)=\left\{\begin{array}{cc} 1\;\; & |S|<S_{c}-3S_{d}\\ \frac{1}{2}\left(1+\tanh\left[\frac{S_{c}-|S|}{S_{d}}\right]\right)\;\; & S_{c}-3S_{d}\leq |S|\leq S_{c}+3S_{d}\\ 0\;\; & S_{c}+3S_{d}<|S| \end{array}\right.$$

Note that *f*_taper_ (*S*_*c*_ − 3*S*_*d*_) ≈ 0.998 and *f*_taper_ (*S*_*c*_ + 3*S*_*d*_) ≈ 0.002. In our simulations in Section 4, we choose *S*_*c*_ = 8 × 10^−3^ and *S*_*d*_ = 5 × 10^−4^. With these values, tapering occurs only in a fraction of the domain, namely near the meridional ridge and in the mixed layer.

## Appendix B Treatment of the Mixed Layer

By definition, potential temperature is approximately homogeneous in the mixed layer. As neutral surfaces appeal to the notion of a strong stratification which inhibits motion in the dianeutral direction, the concept of neutral surfaces does not apply in the mixed layer. That is why the experiments in this study focus on the ocean interior. In the experiments in Section 4, particles are released well below the mixed layer. Still, since neutral surfaces in the Southern Ocean can outcrop to the surface (Marshall & Speer, 2012), particles in our model may be transported to the surface. In Figures 10 and 11, we exclude particles that fall within the mixed layer. Similarly, in the computation of the spurious dianeutral diffusivities in Section 4.6, we exclude particle trajectories that at any point reach depths of −50 m. The actual mixed‐layer, marked by a sharp gradient in potential temperature, lies less deep, but since it varies in space, we use −50 m as a global approximation for computational efficiency.
